# Biofabrication of *Leucas aspera*-Mediated Chitosan–Zinc Oxide Nanocomposites for In Vitro Antioxidant, Antibacterial, Anti-Inflammatory and Wound-Healing Properties

**DOI:** 10.3390/pharmaceutics18030390

**Published:** 2026-03-21

**Authors:** Karuppuchamy Poorani, Manickam Rajkumar, Bhupendra G. Prajapati, Sundar Velmani, Parappurath Narayanan Sudha, Alagarsamy Shanmugarathinam, Himanshu Paliwal

**Affiliations:** 1 Department of Biotechnology, Karpagam Academy of Higher Education (Deemed to be University), Coimbatore 641021, Tamil Nadu, India; 2 Center for Cancer Research, Karpagam Academy of Higher Education (Deemed to be University), Coimbatore 641021, Tamil Nadu, India; 3 Parul Institute of Pharmacy, Faculty of Pharmacy, Parul University, Waghodia, Vadodara 391760, Gujarat, India; bhupen27@gmail.com; 4 Department of Pharmaceutical Engineering, Center for Drug Discovery and Development, Vinayaka Mission’s Kirupanatha Variyar Engineering College, Vinayaka Mission’s Research Foundation (Deemed to be University), Salem 636 308, Tamil Nadu, India; 5 Department of Physiology, Saveetha Dental College and Hospitals, Saveetha Institute of Medical and Technical Sciences (SIMATS), Chennai 602105, Tamil Nadu, India; 6 Department of Pharmaceutical Technology, University College of Engineering, Bharathidasan Institute of Technology, Anna University, Tiruchirappalli 620024, Tamil Nadu, India; 7 Marwadi University Research Center, Faculty of Pharmacy, Marwadi University, Rajkot 360003, Gujarat, India

**Keywords:** nanoparticles, green synthesis, *Leucas aspera*, antibacterial activity, wound healing activity, sustainable biomaterial nanomedicine

## Abstract

**Background/Objectives**: Nanostructured biomaterials based on natural polymers have gained increasing attention in pharmaceutics due to their biocompatibility, multifunctionality, and diverse biomedical applications. This novel study aimed to biofabricate chitosan-doped zinc oxide nanocomposites (CS-ZnONCs) using *Leucas aspera* leaf extract and to evaluate their physicochemical properties and in vitro biomedical performance. **Methods**: CS-ZnONCs were synthesized using *L. aspera* leaf extract through a green precipitation approach, and the resulting nanocomposites were characterized by various spectroscopic techniques. The in vitro antioxidant, antibacterial, and anti-inflammatory activities were evaluated, while wound-healing potential was assessed using L929 fibroblast cell migration assays. **Results**: UV–visible analysis confirmed the formation of CS-ZnONCs, with a characteristic absorption peak at 362 nm, and FTIR spectra indicated the presence of various important functional groups. XRD results demonstrated the crystalline nature of ZnO within the chitosan matrix. Well-dispersed, quasi-spherical nanoparticles with an average size of 44 ± 3.1 nm were identified by HR-TEM, and a positive zeta potential (+9 mV) suggested considerable colloidal stability. CS-ZnONCs showed a high swelling capacity (88 ± 2.75% for 2%) and significant phytocompound release (65.38 ± 2.79% at pH 7.4). The CS-ZnONCs showed significant antioxidant activity (ABTS of 88.19 ± 1.59%), notable antibacterial efficacy against *Staphylococcus aureus* (18.78 ± 0.98 mm) and *Escherichia coli* (17.14 ± 0.96 mm), and significant anti-inflammatory activity (82.12 ± 1.47% membrane stabilization). In vitro biocompatibility and wound-healing assays revealed significant cytocompatibility in Vero cells, with 98.75 ± 1.17% cell viability observed, whereas the fibroblast migration assay demonstrated near-complete wound closure (96.55 ± 6.46%). **Conclusions**: The green-synthesized CS-ZnONCs exhibit favorable physicochemical properties, biocompatibility, and multifunctional biological activities, supporting their potential as a promising sustainable biomaterial nanomedicine for pharmaceutical formulations, wound healing, and regenerative medicine applications.

## 1. Introduction

Nanotechnology has become a transformative field within materials science, providing precise control over nanoparticles at the nanometer scale and contributing to major advancements in biomedical engineering [1]. The development of nanostructured platforms, including polymeric nanoparticles, metal- and metal-oxide-based nanoparticles, nanogels, and hybrid organic–inorganic composites, has been enabled in recent years by the convergence of nanoscience and polymer research. These platforms address critical healthcare challenges, including targeted drug delivery, tissue regeneration, biosensing, and theranostics [2]. Nanoscale systems leverage size-dependent physicochemical properties, including high surface-to-volume ratios, tunable surface chemistry, and stimuli-responsive behavior, to enhance interactions with their biological environments. The biomedical potential of these nanoparticles is further enhanced by functional design strategies that optimize biodegradability, biocompatibility, and selective bioactivity, thereby reducing systemic toxicity and increasing therapeutic efficacy. The size, shape, and surface functionality of nanoparticles can be altered to create highly efficient, multifunctional nanocarriers for a range of biological applications [3,4].

Recent developments in the production and functionalization of metal- and polymer-based nanoparticles have improved their performance in complex physiological environments by enabling precise control over their mechanical characteristics, surface chemistry, size, and shape [5]. Hybrid nanostructures integrate the distinctive physicochemical properties of metals, such as antimicrobial activity, catalytic potential, and electronic characteristics, with the biocompatibility, biodegradability, and tunable functionality of polymers, resulting in multifunctional platforms for biomedical applications [6]. Furthermore, hydrogels, dendrimers, and polymer brushes are examples of macro- and supramolecular constructions that have been shown to replicate extracellular matrices, promote cell adhesion and proliferation, and aid tissue regeneration. The deliberate combination of polymer design and metal nanoparticle engineering at the nanoscale enables the formation of hierarchical architectures that respond to biological signals [7]. Together, these developments demonstrate the critical role that hybrid nanomaterials play in the creation of drug delivery systems, tissue engineering scaffolds, next-generation therapeutics, and antimicrobial strategies. They also emphasize the continued need for interdisciplinary research to optimize their properties further and broaden their biomedical applications [8].

Chitosan (CS), a naturally derived cationic polysaccharide produced by partial deacetylating chitin, possesses distinctive physicochemical and biological properties, including biocompatibility, biodegradability, low toxicity, and intrinsic antimicrobial activity [9,10]. These attributes have established chitosan as a versatile material in nanotechnology-based biomedical research. Reactive amino and hydroxyl groups enable chemical modification and cross-linking, thereby creating chitosan-based hydrogels, films, nanocomposites, and nanoparticles with specific mechanical and biological characteristics. When combined with metal or metal oxide nanoparticles, chitosan forms hybrid nanostructures that exhibit enhanced antimicrobial activity, improved stability, and controlled release [11]. Chitosan–metal nanoparticle composites have been extensively investigated for drug delivery, biosensing, tissue engineering, and gene delivery because they leverage chitosan’s mucoadhesive properties and its ability to enhance cellular uptake [12]. In wound-healing contexts, chitosan–metal nanoparticle dressings accelerate hemostasis and tissue regeneration, provide robust protection against microbial infections, maintain a moist healing environment, stimulate fibroblast proliferation, and promote collagen deposition, thereby offering significant advantages for advanced therapeutic interventions [13,14].

Green synthesis of NPs is a sustainable alternative to conventional physical and chemical methods [15]. This technique reduces metal ions to nanoparticles using biological resources such as plant extracts, microorganisms, and biopolymers. Its advantages include biocompatibility, cost-effectiveness, environmental safety, and scalability [16]. The remarkable physicochemical characteristics of zinc oxide nanoparticles (ZnONPs), including high surface area, semiconducting properties, photostability, and tunable morphology, have attracted considerable attention. These characteristics make ZnONPs suitable for diverse biomedical applications, including antibacterial agents, anticancer therapeutics, anti-inflammatory agents, and wound-healing scaffolds [17]. Phytochemicals act as reducing and capping agents to regulate particle size, shape, and surface chemistry during the green synthesis of ZnONPs using plant extracts, as extensively documented [18]. ZnONPs’ nanoscale size, high surface-to-volume ratio, and surface functional groups are responsible for their increased reactivity and interactions with biological systems [19]. Research has demonstrated that green-synthesized ZnONPs possess significant antimicrobial, antioxidant, and cytotoxic activities against various pathogens and cancer cells. Furthermore, the potential of plant-mediated ZnONPs for biomedical applications has been demonstrated by their ability to accelerate wound healing, promote tissue regeneration, and reduce microbial infections [20,21].

*Leucas aspera* (*L. aspera*) is a medicinal plant recognized for its diverse phytochemical profile, which includes flavonoids, phenolics, alkaloids, terpenoids, and glycosides [22]. Numerous pharmacological characteristics, including antibacterial, antioxidant, and anticancer actions, are exhibited by these bioactive components [23]. The reactive functional groups of these phytochemicals make *L. aspera* a good fit for green nanotechnology, as they enable it to serve as a natural stabilizer, capping agent, and reducing agent during nanoparticle formation. Nanoparticles synthesized using *L. aspera* extracts have demonstrated potential biomedical applications, including enhanced antimicrobial efficacy, tissue regeneration, and drug delivery [24,25]. The use of this plant in nanoparticle synthesis offers several benefits, including low toxicity, cost-effectiveness, environmental sustainability, and ease of preparation. It also eliminates the need for dangerous chemicals commonly used in traditional methods [26]. Comprehensive investigations into the phytochemicals of *L. aspera*, their synergistic interactions, and their mechanistic roles in nanoparticle synthesis remain limited. Although chitosan and ZnONCs have been reported, plant-mediated CS-ZnONCs using *L. aspera* leaf extract have not been explored. In this study, *L. aspera* phytochemicals facilitate a green synthesis strategy by serving as natural reducing and stabilizing agents in the formation of nanocomposites. To the best of our knowledge, this is the first study to use leaf extract from *L. aspera* to describe the synthesis, characterization, and multifunctional biomedical potential of CS-ZnONCs.

The green biofabrication of CS-ZnONPs using leaf extract from *L. aspera* as a natural stabilizing and reducing agent is examined in this study. The resultant nanocomposites were analyzed for their size, shape, surface chemistry, and structural components. The in vitro biological activities of CS-ZnONPs were systematically evaluated, including antioxidant potential, antibacterial efficacy, anti-inflammatory effects, and wound-healing capabilities. This study shows the potential of CS-ZnONPs as safe, efficient, and eco-friendly nanomaterials and highlights their various biomedical applications.

## 2. Materials and Methods

### 2.1. Materials

Zinc nitrate hexahydrate [Zn(NO_3_)_2_·6H_2_O], chitosan, Mueller-Hinton agar (MHA), hydrogen peroxide (H2O_2_), 2,2-diphenyl-1-picrylhydrazyl (DPPH), 2,2′-azino-bis(3-ethylbenzothiazoline-6-sulfonic acid) (ABTS), and 3-(4,5-dimethylthiazol-2-yl)-2,5-diphenyltetrazolium bromide (MTT) were acquired from Sigma-Aldrich Co., St. Louis, MO, USA. The remaining substances and reagents were analytical grade and did not require further purification. Double-distilled water was used for each experiment.

### 2.2. Preparation of Plant Extract

The powdered leaves of *L. aspera* were obtained from Moolihai Wellness Pvt Ltd., Valliyoor, Radhapuram, Tirunelveli 627109, Tamil Nadu, India. To prepare the extract, 100 mL of 70% ethanol and 10 g of plant leaf powder were mixed in a conical flask and swirled at 400 rpm for 12 h at room temperature. Following extraction, plant debris was removed by filtering the mixture through Whatman No. 1 filter paper. The resulting filtrate extract was stored at 4 °C for further use [27,28].

### 2.3. GC-MS Analysis

The phytochemical components of the ethanolic extract of *L. aspera* leaves were identified and characterized by gas chromatography–mass spectrometry (GC-MS). For the analysis, a GC-MS device and a fused-silica capillary column (30 m × 0.25 mm internal diameter, 0.25 μm film thickness) were used. Helium served as the carrier gas and flowed at a constant rate of 1 mL/min. The samples were injected in split mode (10:1) with the injector temperature set to 250 °C. The oven temperature was programmed to increase at 10 °C/min from 60 °C (held for 2 min) to 280 °C, then held at 280 °C for 10 min. Mass spectra were obtained in the m/z range of 40 to 600 using electron ionization at 70 eV. Phytocompounds were identified by matching retention indices reported in the literature and by comparing their mass spectra with the NIST mass spectral collection [29,30].

### 2.4. Biofabrication of CS-ZnONCs

CS-ZnONCs were synthesized using an environmentally friendly green approach employing *L. aspera* leaf extract, following a previously reported method with minor modifications [31,32]. An aqueous solution of zinc nitrate (0.1 M) was prepared and stirred at 50 °C for 2 h using a magnetic stirrer. The zinc precursor solution was continuously stirred, and 20 mL of *L. aspera* leaf extract was added dropwise to promote the biogenic synthesis of ZnO nanoparticles. During synthesis, the reaction pH was adjusted to alkaline conditions (pH ≈ 10) using dilute NaOH, promoting the formation of zinc hydroxide intermediates followed by dehydration into ZnO nanoparticles. Chitosan was dissolved in 1% (*v*/*v*) acetic acid to create a homogenous polymer solution. To ensure homogeneous dispersion, it was added to the ZnO nanoparticle suspension gradually and stirred for 2 h at 60 °C. Sodium tripolyphosphate (TPP) was then introduced as a cross-linking agent to enhance structural stability through ionic interactions between chitosan chains and ZnONPs, yielding a stable, homogeneous nanocomposite dispersion. Following centrifugation at 12,000 rpm for 20 min, the obtained pellet was washed three times with distilled water to remove contaminants and unreacted nitrate ions until a neutral pH (~7) was achieved. The purified nanocomposite suspension was then frozen and lyophilized to obtain a dry powder, which was stored at −20 °C for studies.

### 2.5. Characterization of CS-ZnONCs

The synthesized CS-ZnONCs were examined using several common analytical methods. UV-visible (UV-Vis) spectroscopy in the 200–800 nm range (Shimadzu UV-3600 Plus, Shimadzu Corporation, Kyoto, Japan) confirmed the formation of nanocomposites. Fourier transform infrared (FTIR) spectroscopy was used to determine functional groups in the 400–4000 cm^−1^ region (IRTracer-100, Shimadzu, Tokyo, Japan). X-ray diffraction using Cu Kα radiation was used to analyze the crystalline structure and phase purity (XRD, Malvern Panalytical, Almelo, Netherlands). The surface morphology was examined using Field-emission scanning electron microscopy (FESEM, Thermo Fisher Scientific, Waltham, MA, USA). High-resolution transmission electron microscopy (HR-TEM; Thermo Fisher Scientific, MA, USA) was used to examine the internal morphology and particle size distribution. The elemental composition and purity were assessed by energy-dispersive X-ray spectroscopy (EDX; Ultim Max 40, Oxford Instruments, Oxford, UK). The size distribution and average zeta potential of the CS-ZnONCs were ascertained by dynamic light scattering (DLS, Microtrac, Montgomeryville, PA, USA).

### 2.6. Swelling Properties

The swelling properties of CS-ZnONCs was evaluated using gravimetric analysis [33]. To replicate physiological conditions, precisely weighed biofabricated CS-ZnONCs with an initial dry weight (W_0_) were submerged in phosphate-buffered saline (PBS, pH 7.4) at 37 °C. The samples were removed at pre-arranged intervals (1, 2, 4, 6, 12, 24, and 48 h), carefully blotted with filter paper to remove excess surface liquid, and weighed to calculate the swelled weight (W_t_). The following formula was used to calculate the swelling ratio:

$$Swelling\;ratio\;(\%)=\left[\frac{W_{t}-W_{0}}{W_{0}}\right]\times 100$$

### 2.7. In Vitro Release Properties

The in vitro pH-responsive release of extract-derived phytocompounds from CS-ZnONCs was evaluated using the dialysis bag diffusion method [34]. After being suspended in phosphate-buffered saline (PBS, pH 7.4), a specific quantity of CS-ZnONCs with a molecular weight cut-off of 12–14 kDa was injected into a dialysis membrane. The dialysis bag was submerged in 50 mL of fresh PBS and maintained at 37 ± 0.5 °C with continuous stirring at 100 rpm. At specified intervals, 2 mL of the release medium was removed and replaced with an equivalent volume of fresh PBS to maintain sink conditions. After spectrophotometrically determining the concentration of released bioactive components, cumulative release percentages were computed.

### 2.8. Antioxidant Activity of CS-ZnONCs

#### 2.8.1. DPPH Activity

The antioxidant activity of the CS, ZnONPs, and CS-ZnONCs was assessed using the DPPH free radical scavenging assay. A conventional technique was followed with a few minor adjustments [35]. A freshly made DPPH solution (0.1 mM) in methanol was kept out of direct sunlight. CS-ZnONCs at concentrations ranging from 10 to 200 µg/mL were prepared in distilled water and mixed with the DPPH solution in a 1:1 volume ratio. The mixtures were incubated for 30 min at room temperature in the dark to promote radical scavenging. A UV-visible spectrophotometer was used to measure absorbance at 517 nm. Ascorbic acid was used as the positive control, and the DPPH solution without a sample as the negative control. The DPPH activity percentage was calculated using the following formula:

(1) $$Scavanging\;activity\left(\%\right)=\left[\frac{Absorbance\left(Control\right)-Absorbance\left(Sample\right)}{Absorbance\left(Control\right)}\right]\times 100$$

#### 2.8.2. ABTS Activity

The antioxidant activity of the CS, ZnONPs, and CS-ZnONCs was assessed using the ABTS radical-scavenging assay. A conventional technique was followed with a few minor adjustments [36]. The ABTS radical cation (ABTS^+^•) was produced by reacting a 7 mM ABTS solution with 2.45 mM potassium persulfate. It was then allowed to sit at room temperature in the dark for 12 to 16 h. The resulting ABTS• solution was diluted using phosphate-buffered saline (PBS, pH 7.4). The diluted ABTS^+^• solution was combined with a range of CS-ZnONCs concentrations (10–200 µg/mL) in a 1:1 volume ratio. Following a 6 min incubation period at room temperature in the dark, the absorbance of the combinations at 734 nm was measured using a UV-Visible spectrophotometer. Ascorbic acid served as the positive control, and the ABTS^+^• solution without a sample served as the negative control. The ABTS activity was computed using Equation (1).

#### 2.8.3. H_2_O_2_ Activity

A conventional procedure with a few minor adjustments was used to evaluate the H_2_O_2_-scavenging activity of CS, ZnONPs, and CS-ZnONCs [37]. A freshly prepared 40 mM hydrogen peroxide solution in phosphate-buffered saline (PBS, pH 7.4) was added. Following their production in deionized water, CS-ZnONCs were combined with the hydrogen peroxide solution at varying concentrations (10–200 µg/mL). The sample was incubated at room temperature in the dark for 10 min. After incubation, absorbance was measured at 230 nm using a PBS blank without hydrogen peroxide. The negative control was a hydrogen peroxide solution without a sample, while the positive control was ascorbic acid. The proportion of hydrogen peroxide activity was determined using Equation (1).

### 2.9. Antibacterial Activity

The bacterial strains against which the antibacterial activity of the synthesized CS-ZnONCs was assessed using the agar well diffusion method were obtained by the Institute of Microbial Technology (IMTECH), CSIR, Chandigarh, India, and the MTCC [38,39]. The test organisms comprised Gram-positive bacteria *Staphylococcus aureus* (MTCC 96), *Bacillus cereus* (MTCC 1272), and *Streptococcus oralis* (MTCC 2696), and Gram-negative bacteria *Escherichia coli* (MTCC 443), *Salmonella enterica* serovar Typhimurium (MTCC 98), and *Klebsiella pneumoniae* (MTCC 39). All strains were revived and maintained on Mueller–Hinton agar (MHA) slants and subcultured before use. Sterile saline was used to create fresh bacterial inocula, which were then standardized to a 0.5 McFarland standard (1 × 10^8^ CFU/mL). MHA plates were uniformly inoculated by swabbing the bacterial suspensions across the agar surface. A sterile cork borer was used to drill 5 mm-diameter wells aseptically. Each well received 50 µL of CS-ZnONCs at different doses. The positive control was ampicillin, while the negative control was dimethyl sulfoxide (DMSO). The plates were allowed to diffuse at room temperature for 1 h, then incubated at 37 °C for 24 h. Zones of inhibition were measured in millimeters after incubation.

### 2.10. MIC and MBC Activity

The broth microdilution method was used to determine the minimum inhibitory concentration (MIC) and minimum bactericidal concentration (MBC) of CS-ZnONCs in compliance with CLSI guidelines [40,41]. Overnight cultures of *S. aureus*, *B. cereus*, *S. oralis*, *E. coli*, *S. enterica*, and *K. pneumoniae* were adjusted to approximately 5 × 10^5^ CFU/mL in Mueller–Hinton broth. Serial two-fold dilutions of CS-ZnONCs (0.125–256 µg/mL) were prepared in sterile 96-well microtiter plates. Each well was then filled with 50 µL of the standardized bacterial inoculum. The plates were incubated at 35–37 °C for 18–24 h. *S. oralis* was incubated at 35–37 °C in 5% CO_2_. The MIC was defined as the lowest CS-ZnONCs concentration at which no visible bacterial growth was observed. For MBC determination, aliquots from wells showing no visible growth were spread onto Mueller–Hinton agar plates and incubated under the same conditions. The MBC was defined as the lowest concentration that resulted in a ≥99.9% reduction in the initial bacterial population.

### 2.11. Anti-Inflammatory Activity

The anti-inflammatory effect of CS-ZnONCs was assessed using a slightly modified method of the human red blood cell (HRBC) membrane stabilization experiment described by Adnan et al. [42]. Human blood samples were obtained from the approved blood bank of Karpagam Hospital, Coimbatore, Tamil Nadu, India. Alsever’s solution was prepared by mixing the blood with an equal volume of sterile distilled water containing 2% dextrose, 0.8% sodium citrate, 0.5% citric acid, and 0.42% sodium chloride. The mixture was then centrifuged for ten min at 3000 rpm. After three rounds of washing the resultant packed red blood cells with isotonic saline (0.9% NaCl, pH 7.2), a 10% (*v*/*v*) HRBC suspension was made. The test sample, 1 mL of phosphate buffer (0.15 M, pH 7.4), 2 mL of isotonic saline (0.9%), and 0.5 mL of HRBC suspension were all present in the reaction mixture in different quantities. The positive control used in this study was diclofenac sodium. After 20 min of incubation at 54 °C in a water bath, the test mixtures were centrifuged at 3000 rpm for 10 min. Spectrophotometry at 560 nm was used to measure hemoglobin concentration in the supernatant. The percentage of membrane stabilization and hemolysis was calculated by treating the hemolysis produced in distilled water as 100%.

$$Membrane\;Stabilization\left(\%\right)=\left[\frac{Absorbance\left(Control\right)-Absorbance\left(Sample\right)}{Absorbance\left(Control\right)}\right]\times 100$$

### 2.12. Wound Healing Activity

The synthesized CS-ZnONCs were evaluated for in vitro biocompatibility using Vero epithelial cells and for wound-healing efficacy using L929 fibroblast cells, both obtained from the National Centre for Cell Science (NCCS), Pune, India. Cells were grown in Dulbecco’s Modified Eagle Medium (DMEM) supplemented with 10% fetal bovine serum (FBS) and 1% penicillin–streptomycin solution in a humidified incubator with 5% CO_2_. The culture was maintained at 37 °C [43,44,45]. A sterile 200 µL pipette tip was used to make a straight scratch in the middle of each well to simulate a wound after seeding into 12-well culture plates and attaining confluence. The detached cells were gently washed away using PBS. Following that, cells were subjected to 50 and 100 µg/mL of CS-ZnONCs made in serum-free DMEM. The cells in the control group were untreated. Wound closure was observed at 0, 6, 12, 24, and 48 h using an inverted phase-contrast microscope (Olympus Corporation, Tokyo, Japan), with plates incubated under standard culture conditions. At each time point, the same designated locations were used to capture images of the injured areas. The percentage of wound closure was evaluated to monitor the reduction in wound area over time.

### 2.13. Statistical Analysis

All experiments were conducted in triplicate (n = 3), and the data are expressed as mean ± standard deviation (SD). Statistical analyses were carried out using GraphPad Prism (GraphPad Software, Version 10.6.1, San Diego, CA, USA). To assess group differences, one-way analysis of variance (ANOVA) was followed by Tukey’s post hoc test for multiple comparisons. A *p*-value < 0.05 was set as the cutoff for statistical significance.

## 3. Results and Discussion

### 3.1. GCMS Analysis of L. aspera Leaf Extract

**Figure 1:**
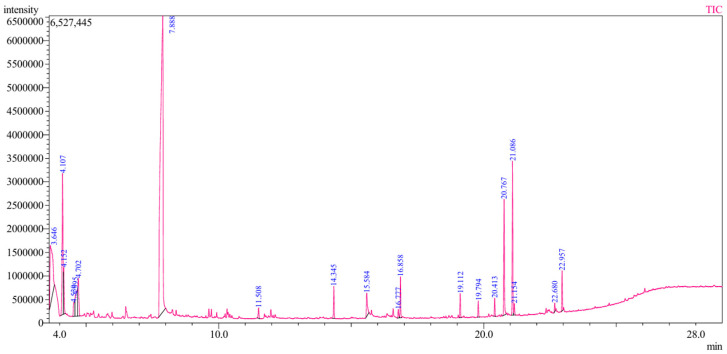
GC–MS analysis of *L. aspera* leaf extract, showing the distribution of phytochemical constituents identified.

GC–MS analysis of *L. aspera* leaf extract identified a diverse array of important phytocompounds (Figure 1). Cyclopentane was the predominant compound, accounting for 49.87% of the peak area, indicating a substantial presence of low-molecular-weight hydrocarbons. Significant quantities of ethane (9.89%), 1,1-diethoxy (8.67%), and 3-methyl-1-butanol (2.04%) were also detected. Fatty acid derivatives accounted for a notable portion of the extract, including hexadecanoic acid ethyl ester (6.15%), hexadecanoic acid methyl ester (0.71%), and octadecanoic acid and its ethyl ester (0.35% and 1.60%, respectively). These fatty acid esters are recognized for their antibacterial and wound-healing properties [46]. Furthermore, long-chain hydrocarbons, including hexadecane (1.68%), octadecane (0.94%), eicosane (0.51%), and tetradecane (1.35%), were identified and are commonly linked to membrane-active antimicrobial effects [47]. Aromatic ester compounds, such as 1,2-benzenedicarboxylic acid derivatives (diethyl, butyl, and bis(2-methylpropyl) esters; collectively approximately 7.5%), may contribute to the stabilization of bioactive formulations (Table 1). GC–MS analysis revealed a diverse phytochemical profile of the ethanolic extract, indicating the presence of several plant-derived metabolites. However, some detected compounds may be due to analytical artifacts or analytical interference. Collectively, the presence of fatty acid esters, alcohols, and aromatic compounds provides a biochemical basis for the observed antioxidant, antibacterial, anti-inflammatory, and wound-healing activities, supporting its application in the biofabrication of functional nanocomposites [48,49].

### 3.2. UV-Vis Spectroscopy

**Figure 2:**
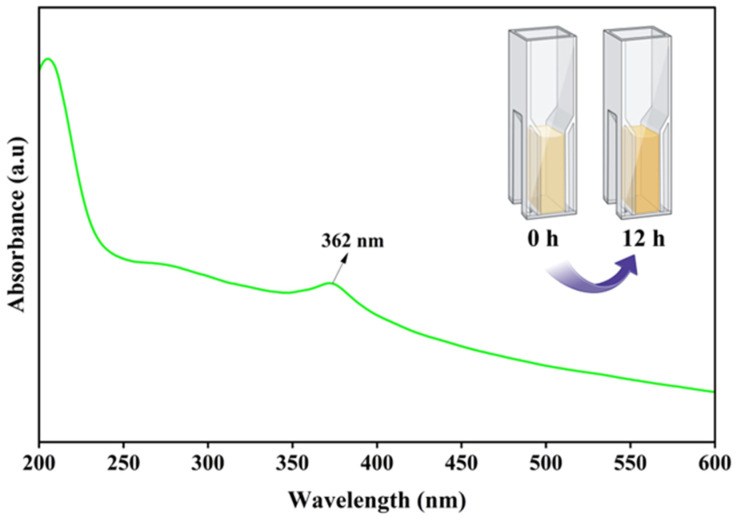
UV–Vis absorption spectrum of CS-ZnONCs synthesized using *L. aspera* leaf extract.

The synthesis of CS-ZnONCs using *L. aspera* leaf extract was initially confirmed using UV-visible spectroscopy (Figure 2). ZnONPs are characterized by a distinct absorption band centered at approximately 362 nm in the resulting UV-Vis spectra. This band represents intrinsic band-gap absorption from the valence to the conduction band. The presence of this absorption peak indicates that ZnONPs are effectively produced within the chitosan matrix. The absorption edge observed at approximately 362 nm is slightly shifted relative to that of bulk ZnO, likely due to the particles’ nanoscale dimensions and interactions between ZnO nanoparticles and chitosan polymer chains [50].

The wide absorption band also indicates a highly restricted particle-size distribution and effective stabilization of CS-ZnONCs by the chitosan and phytochemicals present in the *L. aspera* leaf extract. Plant-derived biomolecules, including flavonoids, phenolics, and proteins, likely functioned as reducing and capping agents during synthesis, thereby preventing agglomeration and enhancing nanoparticle stability [51]. A previous study reported that ZnONPs synthesized from *Casuarina equisetifolia* exhibited a UV-Vis absorption peak at approximately 360 nm, confirming nanoparticle formation [52]. Another study on the biosynthesis of ZnONPs using *Calotropis gigantea* leaf extract reported a UV-Vis absorption peak comparable to that of ZnONPs, thereby confirming their formation [53].

### 3.3. FTIR Analysis

**Figure 3:**
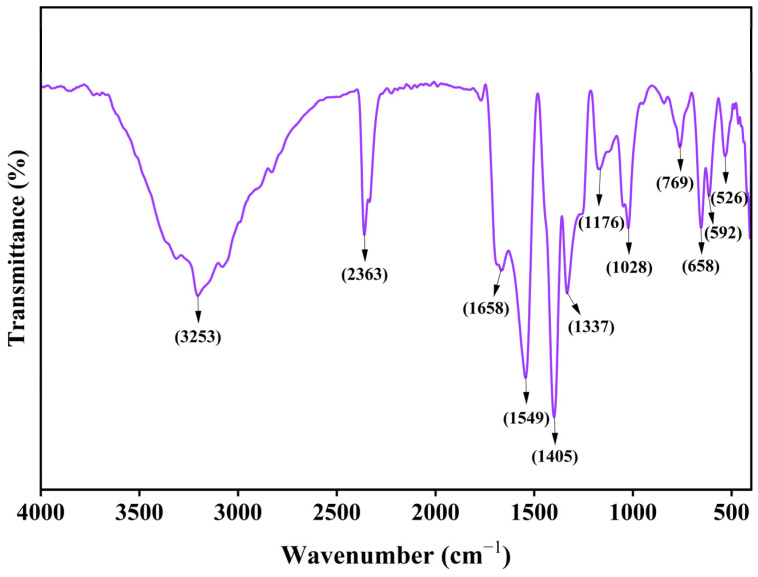
FTIR spectrum analysis of synthesized CS-ZnONCs, showing characteristic functional groups.

FTIR spectroscopy was used to identify the functional groups responsible for the biofabrication and stability of CS-ZnONCs made from *L. aspera* leaf extract (Figure 3). A sizable absorption band in the FTIR spectrum, centered at roughly 3253 cm^−1^, represents the stretching vibrations of the amine (–NH) and hydroxyl (–OH) groups. This band, characteristic of chitosan and plant-derived polyphenolic compounds, indicates strong hydrogen-bonding interactions and confirms the involvement of biomolecules in nanoparticle stabilization [54]. The absorption band at 2363 cm^−1^ is caused by both ambient CO_2_ adsorption and modest C–H stretching vibrations, which are frequently observed in biopolymer-based nanocomposites [55]. Chitosan’s amide I (C=O stretching) vibrations are correlated with a strong peak at 1658 cm^−1^, whereas amide II (N–H bending) vibrations are linked to a band at 1549 cm^−1^. Shifts in these amide bands relative to pure chitosan suggest strong coordination interactions between ZnONPs and chitosan’s amino groups [56].

The C–N stretching or symmetric stretching of carboxylate groups is responsible for the peak at 1405 cm^−1^, which further bolsters the interaction of Zn^2+^ ions with functional groups in chitosan and *L. aspera* phytochemicals. The absorption at 1176 cm^−1^ is caused by C–O–C stretching vibrations of the chitosan backbone, while the band indicates H bending vibrations at 1337 cm^−1^ [57]. The peak confirms the presence of polysaccharide structures at 1028 cm^−1^, which is associated with C–O stretching vibrations. Characteristic bands confirmed the creation of ZnONPs within the chitosan matrix at 769, 658, 592, and 526 cm^−1^ in the low-wavenumber range. These bands are attributable to Zn–O stretching vibrations. The presence of these Zn–O bands, together with shifts in the functional group peaks of chitosan and plant extract, demonstrates the successful formation of CS-ZnONCs through strong interfacial interactions. The synergistic interactions facilitated the development of a stable and biocompatible CS-ZnONCs system, thereby enhancing its suitability for biomedical applications. A recent study reported that biosynthesized CS–ZnONPs using *Miswak extracts* showed results similar to those reported in the present study [58]. Another study on ZnO/chitosan nanocomposites reported similar results, in good agreement with the present findings [59].

### 3.4. XRD Analysis

**Figure 4:**
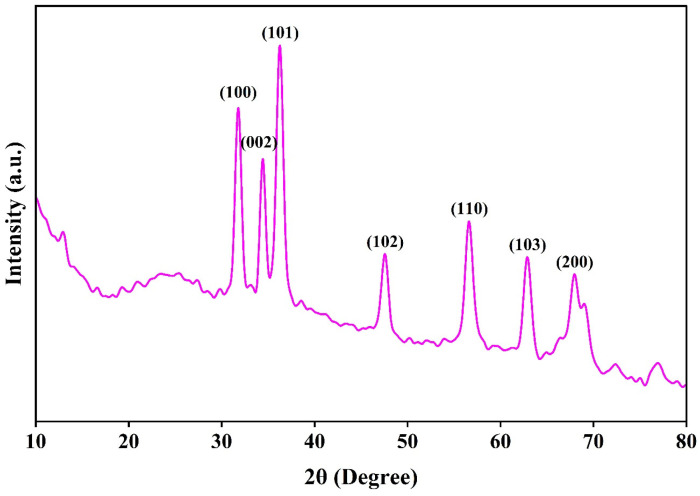
XRD pattern of synthesized CS-ZnONCs using *L. aspera* leaf extract, showing characteristic diffraction peaks corresponding to the crystalline ZnO phase.

The crystalline structure and phase composition of CS-ZnONCs were examined using XRD. The XRD pattern displays a broad diffraction band centered at approximately 2θ ≈ 23°, indicative of the semicrystalline nature of chitosan and reflecting intermolecular hydrogen bonding and ordered chain arrangement within the polymer backbone (Figure 4). This broad peak demonstrates the effective incorporation of chitosan into the nanocomposite matrix. In addition to the chitosan-associated reflection, several sharp, intense diffraction peaks are observed at 2θ values of 31.82°, 34.23°, 36.52°, 47.64°, 56.74°, 62.98°, and 67.52°. These peaks correspond to the (100), (002), (101), (102), (1103), and (200) planes of hexagonal wurtzite ZnO, respectively (JCPDS card No. 36-1451). The effective synthesis of CS-ZnONCs is confirmed by the simultaneous existence of the broad chitosan peak and the sharp ZnO peaks [56,60].

The most intense reflection at 36.0°, corresponding to the (101) plane, suggests preferential growth orientation and high crystallinity of ZnONPs within the chitosan matrix [52]. Additional diffraction peaks at 31.82° (100) and 34.0° (002) further confirm the formation of the hexagonal wurtzite structure. The higher-angle reflections at 47.0°, 56.0°, 62.0°, and 67.0° indicate well-developed crystal planes and structural stability of the ZnO nanophase [61]. The slight broadening and reduced intensity of ZnO diffraction peaks in CS-ZnONCs, compared to bulk ZnO, are attributed to the nanoscale size of ZnO crystallites, their interaction with chitosan chains, and effective capping by phytochemicals from the *L. aspera* leaf extract. Notably, no additional peaks associated with impurity phases or unreacted precursors were observed, indicating the high purity of the synthesized nanocomposites. These XRD analyses confirm the effective integration of crystalline ZnONPs into the semi-crystalline chitosan matrix. A recent study reported that ZnONPs synthesized using *Plumeria leaf* extract yielded results comparable to those observed in the current study [62]. Another study reported that *Juglans regia* leaf extract-mediated chitosan-ZnONPs exhibited XRD patterns similar to those observed in the present study [63].

### 3.5. FESEM-EDX Analysis

**Figure 5:**
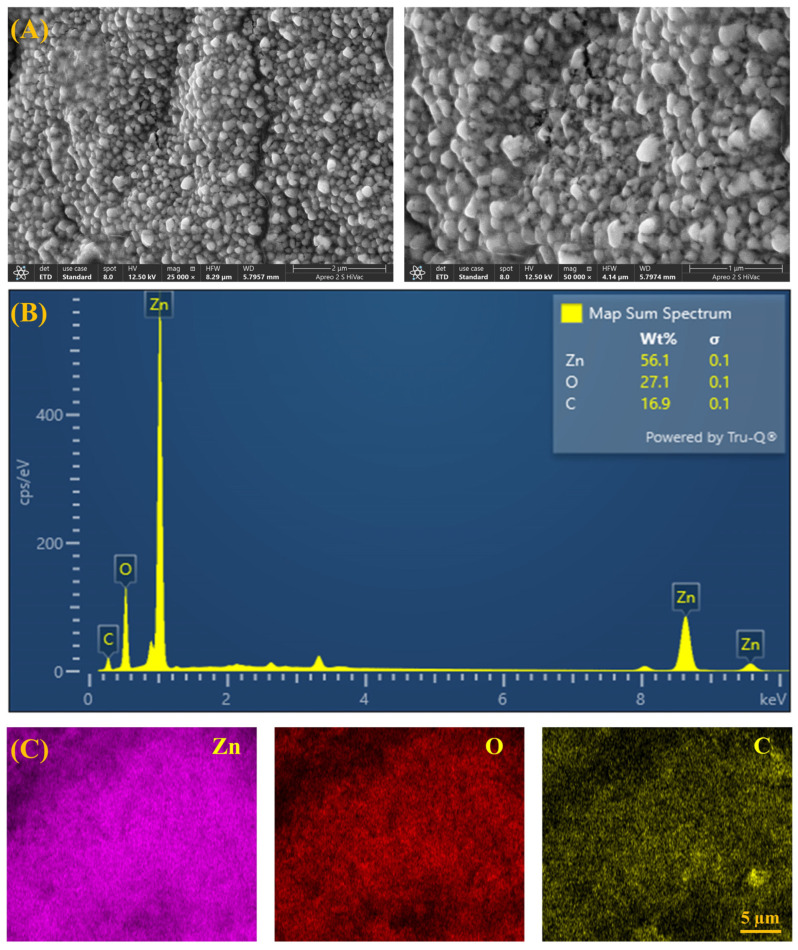
(**A**) FESEM images showing the surface morphology of synthesized CS-ZnONCs using *L. aspera* leaf extract at different magnifications. (**B**) EDX spectrum confirming the elemental composition of Zn, O, and C. (**C**) Elemental mapping images illustrating the uniform distribution of Zn, O, and C within the nanocomposites.

The surface morphology of CS-ZnONCs prepared from an *L. aspera* extract was investigated using FESEM (Figure 5A). The resulting micrographs show a densely packed nanostructured surface, consisting of quasi-spherical to irregular ZnONPs uniformly embedded in the chitosan matrix [64]. FESEM particle size analysis confirms successful nanoscale synthesis, revealing that CS-ZnONCs have an average size of 57 ± 4.3 nm and a size range of 40 to 75 nm. Strong intermolecular interactions between ZnO nanoparticles and the chitosan polymer network, such as hydrogen bonds and electrostatic forces, likely lead to minor agglomeration in some regions [65].

The elemental composition of the CS-ZnONCs was verified by the EDS analysis (Figure 5B). The EDS spectrum displays distinct peaks for Zn, O, and C, with weight percentages of 56.1%, 27.1%, and 16.9%, respectively. The elevated Zn and O levels verify the synthesis of ZnO, while the carbon signal is ascribed to the chitosan backbone and phytochemical residues from *L. aspera*. The absence of additional peaks suggests that the nanocomposites are extremely pure [66,67].

Elemental mapping analysis (Figure 5C) further demonstrates the homogeneous distribution of Zn, O, and C throughout the composite matrix, confirming the uniform incorporation and stabilization of ZnO nanoparticles within the chitosan framework. Recent research on CS-ZnONCs has reported structural morphologies and nanoparticle sizes comparable to those observed in the present study [68]. Furthermore, another study found that the CS/Zn nanocomposite was spherical, with nanoparticle sizes reported by Asghar et al. [59].

### 3.6. HRTEM and DLS-Zeta Potential Analysis

**Figure 6:**
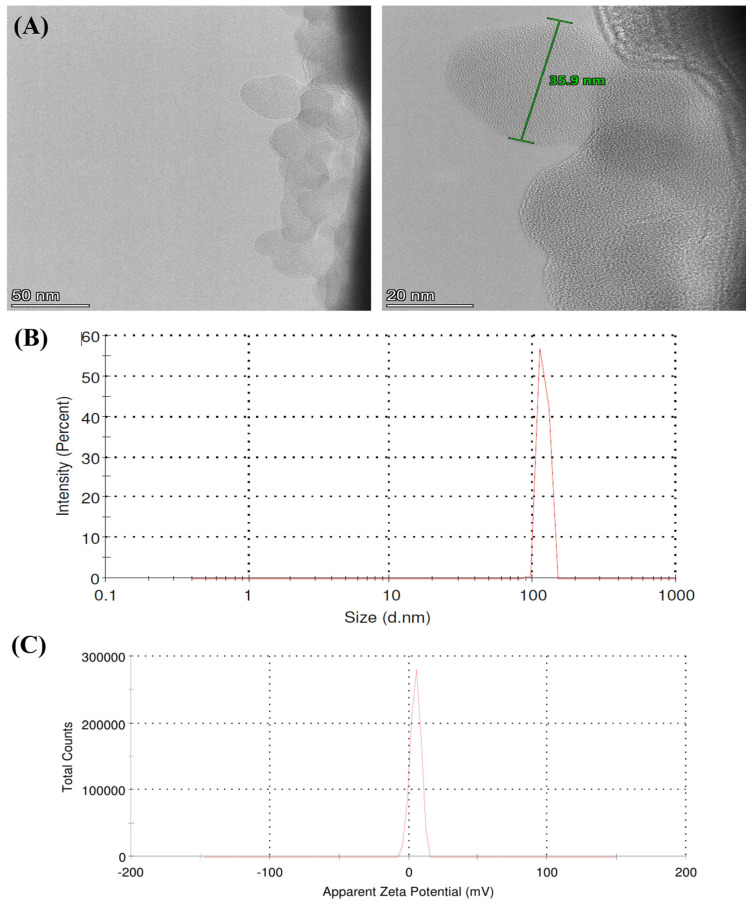
(**A**) HR-TEM analysis images of synthesized CS-ZnONCs using *L. aspera* leaf extract, showing nanoscale morphology. (**B**) DLS analysis and (**C**) Zeta potential distribution indicating the colloidal stability and surface charge of the nanocomposites.

The morphology and particle analysis size of the CS-ZnONCs were examined using HR-TEM, as shown in Figure 6A. The HR-TEM images show well-defined ZnONPs, ranging from nearly spherical to slightly irregular, uniformly distributed within the chitosan matrix. These nanoparticles exhibit good dispersion and minimal agglomeration, suggesting effective stabilization by both the chitosan polymer and the plant extract’s phytochemical constituents [63]. The particle size analysis of the HR-TEM micrographs indicates that the nanoscale CS-ZnONCs were successfully synthesized, with an average size of 44 ± 3.1 nm and a size range of 36–52 nm. The distinct particle boundaries and consistent image contrast further support the efficient encapsulation of ZnONPs within the biopolymeric framework [63].

The hydrodynamic size distribution and colloidal behavior of the synthesized CS-ZnONCs were evaluated using DLS analysis. At about 110 nm, the DLS profile displayed a single, narrow peak (Figure 6B), indicating uniform nanoparticle formation. This monomodal distribution suggests that ZnONPs are effectively stabilized within the chitosan matrix, which limits aggregation. Since HR-TEM measures the dry core, and DLS analyzes the hydrated diameter, polymer coating, and solvent layer, the hydrodynamic size recorded by DLS was greater than the particle size observed in HR-TEM [69]. These findings confirm successful biofabrication and stable dispersion of CS-ZnONCs in aqueous suspension. Chitosan provides steric stabilization through polymer chain interactions, supporting suspension stability even with moderate surface charge. Similar DLS size-distribution results have been reported in recent studies on chitosan-based coatings incorporating ZnONPs [70].

Zeta potential analysis was used to further characterize the surface charge of CS-ZnONCs (Figure 6C). The zeta potential showed a narrow peak at +9 mV, indicating moderate electrostatic stability. This positive charge results from protonated amino groups (–NH_3_^+^) in chitosan, which enhance dispersion stability and limit aggregation. These surface properties are beneficial for biomedical applications, as they support interaction with cellular membranes and maintain colloidal integrity. Together, DLS, HR-TEM, and zeta potential analyses confirm the successful green synthesis of CS-ZnONCs with controlled nanoscale dimensions and appropriate surface properties using *L. aspera* extract. These investigations are consistent with earlier work on CS/Zn-based nanocomposites [71].

### 3.7. Swelling and In Vitro Release Properties

**Figure 7:**
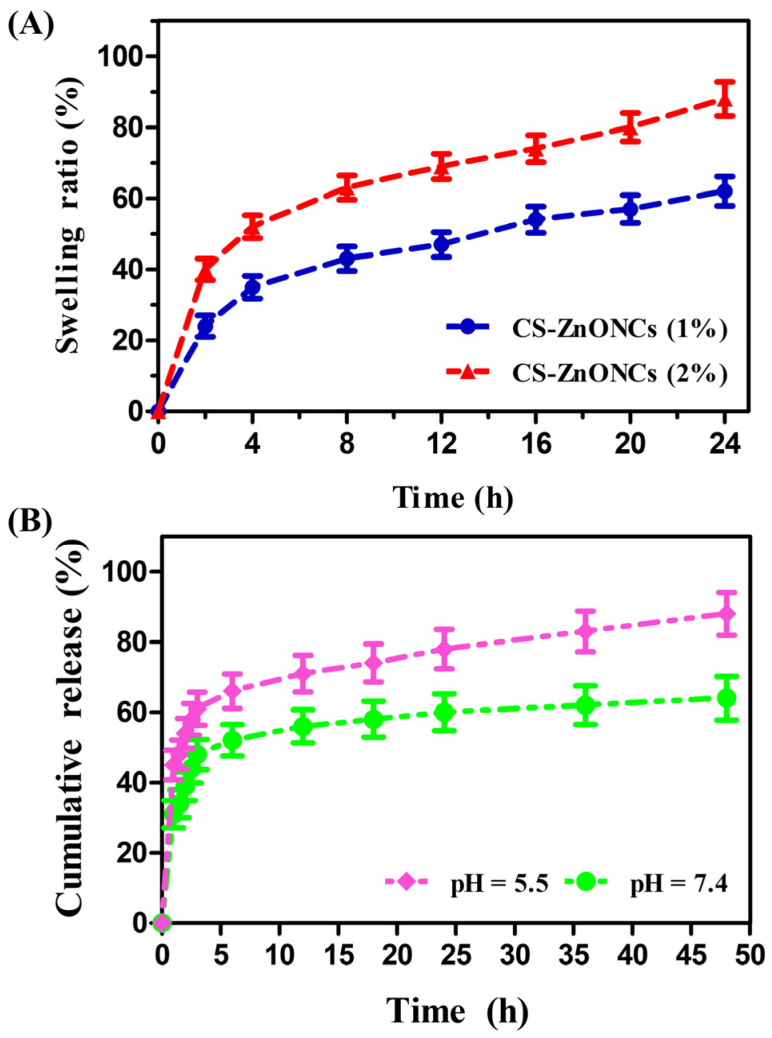
(**A**) Time-dependent swelling ratio of CS-ZnONCs (1% and 2%) showing a progressive increase in swelling over time, with the 2% formulation exhibiting a higher swelling capacity compared to the 1% formulation. (**B**) In vitro pH-responsive release profile of plant-derived phytoconstituents from CS-ZnONCs at pH 5.5 and 7.4, showing enhanced release under acidic conditions (pH 5.5) and comparatively controlled release at physiological pH (7.4).

The swelling behavior of CS-ZnONCs nanocomposites was evaluated over 24 h to assess their hydration capacity and structural stability for biomedical applications. Time-dependent swelling was observed in both formulations, with rapid initial water uptake and a slow approach to equilibrium. After 24 h, the CS-ZnONCs (1%) formulation showed a swelling ratio of 59 ± 2.17%, whereas the CS-ZnONCs (2%) formulation exhibited significantly higher swelling, reaching 88 ± 2.75% (Figure 7A). The enhanced swelling observed in the 2% nanocomposite is likely due to increased incorporation of ZnO NPs, thereby increasing hydrophilicity and promoting the formation of a more porous polymer network [72,73]. This increased capacity for swelling suggests improved diffusion pathways and exudate absorption, which are advantageous for drug delivery and wound-dressing applications.

The in vitro pH-responsive release behavior of extract-derived phytocompounds from CS-ZnONCs was evaluated under different pH conditions. As shown in Figure 7B, both formulations exhibited an initial controlled release, followed by continuous release over 48 h. The cumulative release of phytocompounds reached 90.47 ± 3.58% at an acidic pH (5.5), whereas a comparatively lower, controlled release of 65.38 ± 2.79% was observed at pH 7.4. The higher release at acidic pH may be attributed to increased polymer matrix relaxation and enhanced diffusion of phytocompounds. This prolonged and pH-dependent release pattern indicates that CS-ZnONCs nanocomposites can provide controlled drug delivery, which may help reduce systemic toxicity while maintaining effective therapeutic concentrations at wound sites [74,75]. Recent studies have shown that pH-responsive nanocarriers enable controlled release, which is closely related to the present study [76]. Overall, the swelling and phytocompound-release results demonstrate the potential of CS-ZnONCs as multifunctional biomaterials for antimicrobial therapy and wound-healing applications, highlighting their excellent water-absorption capacity and controlled release properties.

### 3.8. Antioxidant Activity

**Figure 8:**
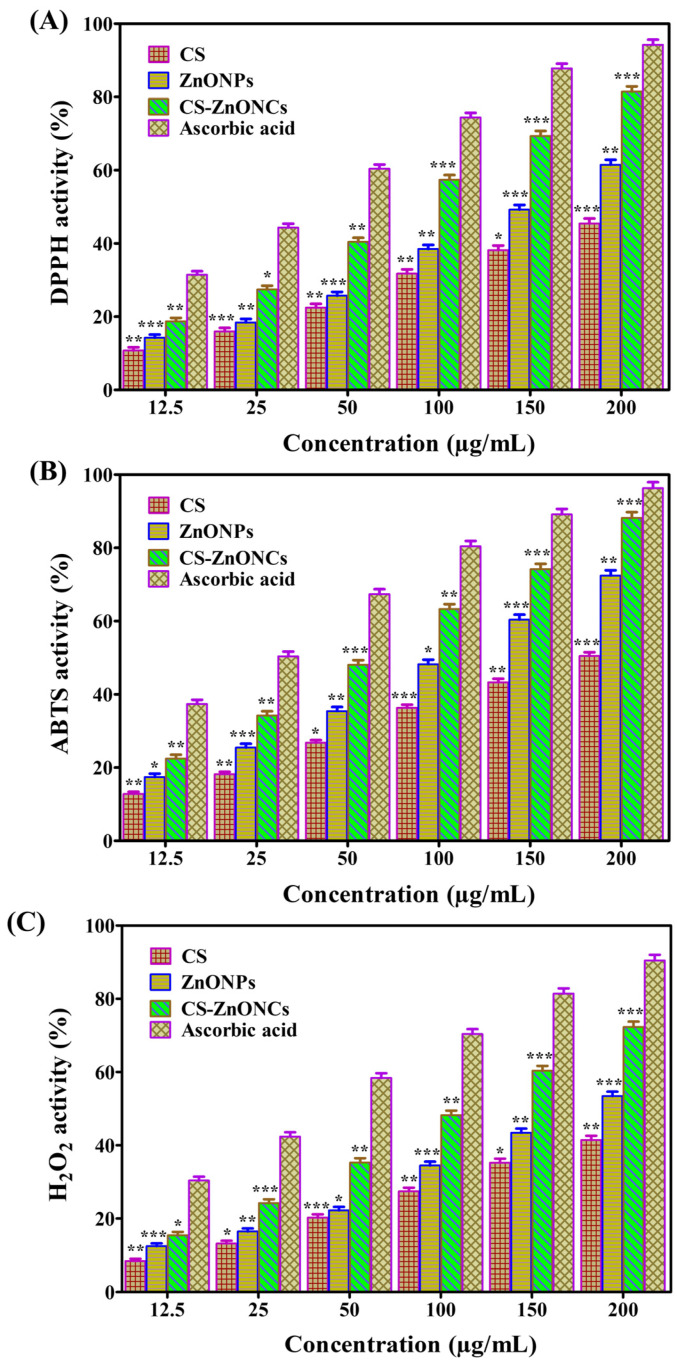
Antioxidant activity of synthesized CS, ZnONPs, and CS-ZnONCs evaluated using (**A**) DPPH, (**B**) ABTS, and (**C**) H_2_O_2_ radical scavenging assays at different concentrations, with ascorbic acid used as the standard. The data are expressed as mean ± SD (n = 3); statistical significance was established at * *p* < 0.05, ** *p* < 0.01, and *** *p* < 0.001 when compared to the control.

The antioxidant capacity of CS-ZnONCs produced using *L. aspera* extract was assessed using the DPPH, ABTS, and H_2_O_2_ radical scavenging assays. The PC was ascorbic acid. In all tests, CS-ZnONCs showed a concentration-dependent rise in radical-scavenging activity, indicating strong antioxidant activity. In the DPPH assay, CS exhibited 45.61 ± 1.18% scavenging activity, ZnONPs showed 61.47 ± 1.28%, while CS-ZnONCs demonstrated significant scavenging activity of 81.47 ± 1.42%, and ascorbic acid showed 94.25 ± 1.60% inhibition at a concentration of 200 µg/mL (Figure 8A). The IC_50_ values further supported these results, with CS showing 216.74 µg/mL, ZnONPs 151.63 µg/mL, and CS-ZnONCs 92.24 µg/mL, compared with 41.89 µg/mL for ascorbic acid, indicating the enhanced free-radical scavenging efficiency of the nanocomposite [77].

In the ABTS assay (Figure 8B), CS exhibited 50.47 ± 1.12% scavenging activity, ZnONPs showed 72.43 ± 1.44%, while CS-ZnONCs demonstrated a maximum scavenging activity of 88.19 ± 1.59% when compared to ascorbic acid, which exhibited 96.31 ± 1.75% inhibition. The IC_50_ values were 186.09 µg/mL for CS, 113.71 µg/mL for ZnONPs, and 74.12 µg/mL for CS-ZnONCs, compared with ascorbic acid 20.29 µg/mL, indicating an enhanced electron-donating and radical-scavenging capacity of the nanocomposite [78]. The H_2_O_2_ activity (Figure 8C) further confirmed the antioxidant potential, where compared with PC, the CS exhibited 41.52 ± 1.09% inhibition, ZnONPs showed 53.92 ± 1.25%, and CS-ZnONCs showed 72.36 ± 1.42% scavenging activity, and ascorbic acid showed 90.47 ± 1.56%. Ascorbic acid showed an IC_50_ value of 48.51 µg/mL, while the values for CS, ZnONPs, and CS-ZnONCs were 240.95 µg/mL, 180.10 µg/mL, and 115.20 µg/mL, respectively. The synergistic interactions among ZnONPs, chitosan’s functional groups, and bioactive compounds from *L. aspera* are responsible for this antioxidant activity. These interactions together improve the scavenging of reactive oxygen species [79].

A synergistic mechanism involving the hydroxyl and amino groups of chitosan donating electrons or hydrogen atoms, ZnONPs’ surface-defect-mediated redox activity, and the presence of phenolic and flavonoid compounds from *L. aspera* extract is responsible for the observed antioxidant activity of CS-ZnONCs. Together, these elements support the stabilization of radicals and the neutralization of ROS, thereby improving the nanocomposites’ antioxidant performance [80,81]. DPPH (76.56%) and ABTS (77.24%) assays showed significant antioxidant activity in recent studies on chitosan-doped ZnO prepared from *Annona muricata* [82]. An aqueous extract of *Azadirachta indica* was used to prepare zinc- and chitosan-based nanoparticles, yielding results consistent with those of the current investigation [83].

### 3.9. Antibacterial Activity

**Figure 9:**
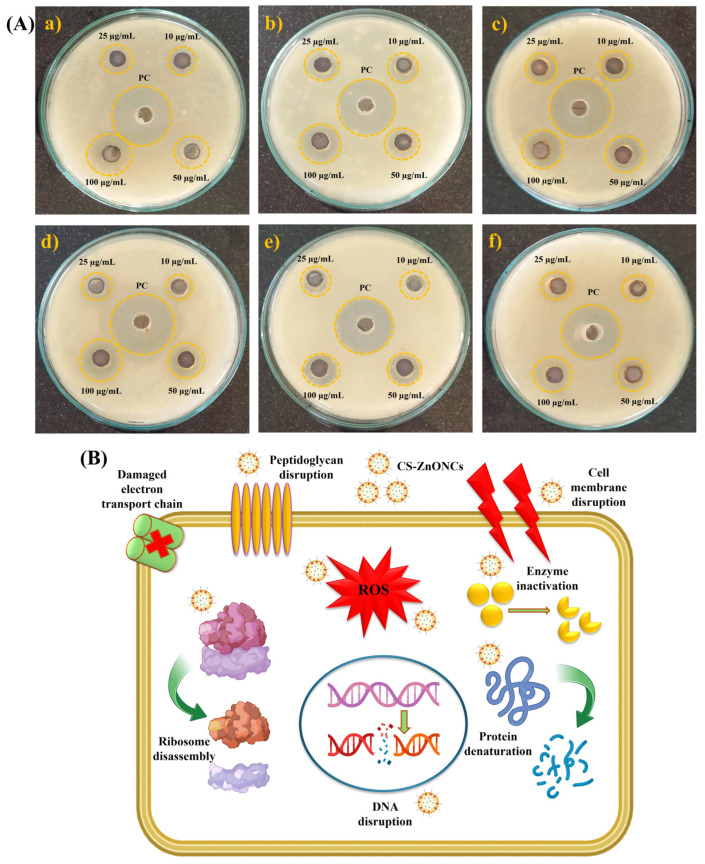
(**A**) Antibacterial activity of synthesized CS-ZnONCs using *L. aspera* leaf extract was evaluated by the agar well diffusion method against (**a**) *S. aureus*, (**b**) *B. cereus*, (**c**) *S. oralis*, (**d**) *E. coli*, (**e**) *S. enterica*, and (**f**) *K. pneumoniae* at various concentrations (10–100 µg/mL), with a positive control (PC). (**B**) The suggested antibacterial mechanism of CS-ZnONCs involves the production of ROS, disruption of the peptidoglycan layer and cell membrane, inactivation of enzymes, denaturation of proteins, ribosomal damage, and DNA disruption, resulting in bacterial cell death.

The antibacterial activity of CS-ZnONCs against various pathogenic bacteria was assessed using the agar well diffusion method (Figure 9A). At the highest measured concentration of 100 µg/mL, CS-ZnONCs demonstrated notable antibacterial activity, with inhibition zones measuring 17.78 ± 0.98 mm for *S. aureus*, 15.91 ± 0.84 mm for *B. cereus*, and 15.42 ± 0.79 mm for *S. oralis*. For Gram-negative strains, inhibition zones were 17.14 ± 0.96 mm for *E. coli*, 16.78 ± 0.91 mm for *S. enterica*, and 15.83 ± 0.89 mm for *K. pneumoniae*. While the PC produced slightly larger inhibition zones (19–22 mm), the antibacterial efficacy of CS-ZnONCs at 100 µg/mL was statistically significant compared to lower concentrations (Table 2) [32]. A limitation of this study is that the antibacterial activity of CS, ZnO, and the plant extract requires further evaluation, and future studies should focus on investigating their individual contributions and the underlying molecular mechanisms responsible for the observed antibacterial effects. Previous studies have also shown that folic acid–modified CS-ZnONPs possess significant antibacterial activity [84].

The synergistic interaction between ZnONPs and the chitosan matrix is responsible for the increased antibacterial activity of CS-ZnONCs at 100 µg/mL. Aouadi et al. [85] reported that the positively charged amino groups in chitosan interact electrostatically with the negatively charged bacterial cell surfaces, disrupting membrane integrity, increasing permeability, and allowing internal components to flow out. This instability facilitates the entry of ZnONPs into bacterial cells. ZnONPs simultaneously generate ROS, including superoxide anions and hydroxyl radicals, which induce oxidative stress in bacterial cells. Lipid peroxidation, protein denaturation, enzyme deactivation, and DNA fragmentation are all consequences of ROS-mediated damage [86]. Additionally, interference with the electron transport chain disrupts ATP synthesis, resulting in metabolic failure. These combined effects also promote ribosomal disassembly and inhibit protein synthesis (Figure 9B) [87]. Collectively, these multi-targeted mechanisms overwhelm bacterial defense systems, leading to bacterial cell death and accounting for the broad-spectrum antibacterial efficacy of CS-ZnONCs.

### 3.10. MIC and MBC Activity

The MIC and MBC against several pathogenic bacteria were determined to evaluate the antibacterial efficacy of CS-ZnONCs. The results are described in Table 3. The nanocomposite showed significant antibacterial activity against both Gram-positive and Gram-negative strains. *S. aureus* exhibited the lowest MIC (10 µg/mL) and an MBC of 25 µg/mL, indicating strong susceptibility. *B. cereus* and *E. coli* showed moderate activity, with MIC and MBC values of 25 µg/mL and 50 µg/mL, respectively. *S. oralis*, *S. enterica,* and *K. pneumoniae* had higher MIC and MBC values (50 and 100 µg/mL), indicating lower sensitivity to the nanocomposite [88]. The CS-ZnONCs MIC and MBC values reported in the current study were closer to those reported in earlier studies [89].

Differences in bacterial cell wall structure are likely responsible for differences in antibacterial susceptibility. Strong interactions between positively charged chitosan and the thick peptidoglycan layer of Gram-positive bacteria enhance their antibacterial activity. Gram-negative bacteria, on the other hand, have a lipopolysaccharide outer membrane that acts as a permeability barrier, restricting the entry of nanomaterials [90]. The enhanced antibacterial performance of CS-ZnONCs is due to the synergistic effect of chitosan and zinc oxide nanoparticles. Chitosan disrupts bacterial membranes via electrostatic interactions, thereby increasing permeability and causing leakage. ZnONPs generate reactive oxygen species and release Zn^2+^ ions, inducing oxidative stress and cell death [91]. The MIC and MBC results confirm that CS-ZnONCs have strong antibacterial potential against diverse pathogens, supporting their use as effective antimicrobial agents in biomedical and pharmaceutical applications.

### 3.11. Anti-Inflammatory Activity

**Figure 10:**
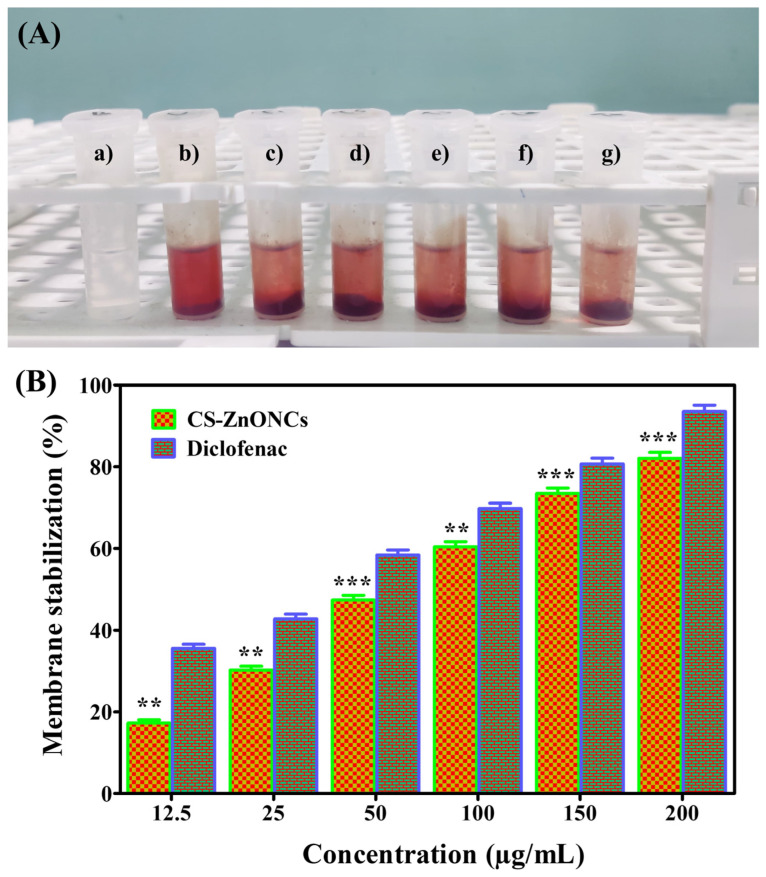
(**A**) Anti-inflammatory activity of CS-ZnONCs synthesized using *L. aspera* leaf extract at different concentrations: (**a**) blank, (**b**) 12.5, (**c**) 25, (**d**) 50, (**e**) 100, (**f**) 150, and (**g**) 200 μg/mL, showing visible changes in the reaction mixtures during membrane stabilization. (**B**) Anti-inflammatory activity of CS-ZnONCs evaluated by the membrane stabilization assay at various concentrations, compared with diclofenac as the standard drug. The data are expressed as mean ± SD (n = 3); statistical significance was established at ** *p* < 0.01, and *** *p* < 0.001 when compared to the control.

The HRBC membrane stabilization experiment was used to evaluate the anti-inflammatory activity of CS-ZnONCs made from *L. aspera* extract, as shown in Figure 10. Visual inspection of the HRBC assay revealed a clear reduction in hemolysis in the presence of CS-ZnONCs, indicating effective stabilization of the erythrocyte membrane (Figure 10A). For CS-ZnONCs, quantitative analysis showed a concentration-dependent increase in membrane stability. CS-ZnONCs at 200 µg/mL exhibited a maximum membrane stabilization of 82.12 ± 1.47%, whereas the positive control achieved 93.57 ± 1.76%. The calculated IC_50_ value for CS-ZnONCs was 83.99 µg/mL, higher than that of diclofenac (43.73 µg/mL), indicating strong but comparatively weak anti-inflammatory potency relative to the standard drug (Figure 10B). Statistical analysis confirmed that the membrane-stabilizing effects of CS-ZnONCs were significantly greater than those at lower concentrations [92].

The ability of CS-ZnONCs to stabilize cellular and lysosomal membranes, thereby preventing the release of inflammatory mediators, including proteases and phospholipases, is the main mechanism by which they reduce inflammation. The polycationic chitosan interacts with membrane phospholipids and proteins, enhancing membrane integrity. At the same time, ZnONPs and bioactive phytochemicals from *L. aspera* contribute to reactive oxygen species scavenging and inhibition of lipid peroxidation [93,94]. These combined effects reduce membrane damage and inflammatory responses, supporting the therapeutic potential of CS-ZnONCs. An investigation on ZnONPs mediated by *Pentatropis capensis* revealed notable anti-inflammatory efficacy, with an IC_50_ value of 79.86 μg/mL for stabilizing HRBC membranes [95]. Another study reported that *Myristica fragrans* extract–ZnONPs exhibited strong anti-inflammatory activity in HRBC membrane stabilization assays [96]. These results align with those found in the current investigation.

### 3.12. Cytotoxic Activity

**Figure 11:**
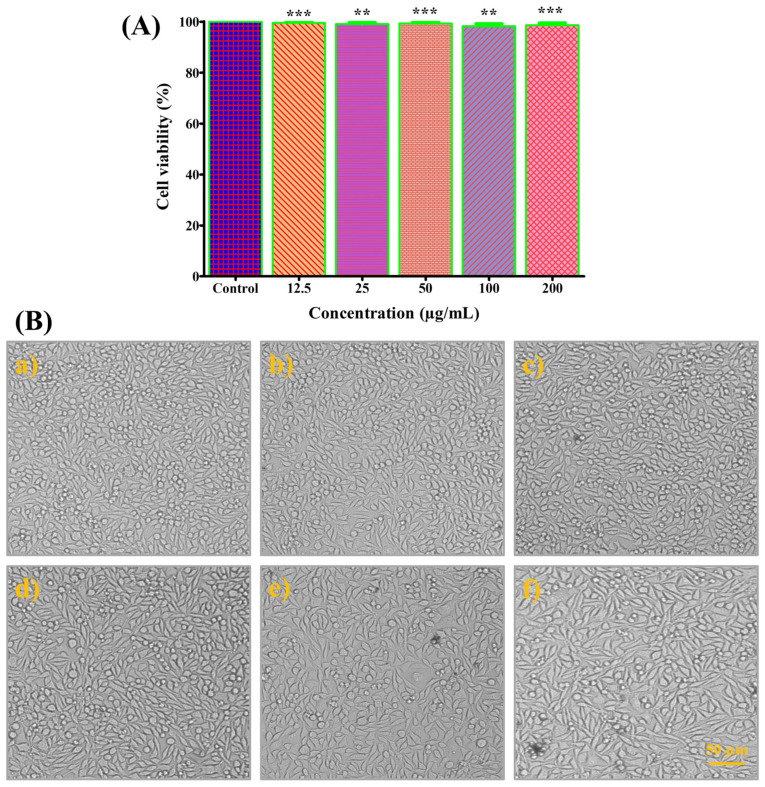
Cytotoxicity evaluation of CS-ZnONCs in the Vero cell line. (**A**) Cell viability of Vero cells after treatment with different concentrations of CS-ZnONCs (12.5–200 µg/mL) compared with the untreated control. Data represent the percentage of viable cells, indicating high biocompatibility of CS-ZnONCs toward normal cells. The data are expressed as mean ± SD (n = 3); statistical significance was established at ** *p* < 0.01, and *** *p* < 0.001 when compared to the control. (**B**) Phase-contrast microscopic images showing the morphology of Vero cells after exposure to CS-ZnONCs at various concentrations: (**a**) Control, (**b**) 12.5 µg/mL, (**c**) 25 µg/mL, (**d**) 50 µg/mL, (**e**) 100 µg/mL, and (**f**) 200 µg/mL. Cells retained normal morphology and confluency with no significant structural damage, confirming the low cytotoxic effect of CS-ZnONCs. Scale bar: 50 µm.

The cytotoxicity of CS-ZnONCs was assessed using the Vero cell line to evaluate their safety and biocompatibility for biomedical applications. As shown in Figure 11A, Vero cells treated with 12.5–200 µg/mL CS-ZnONCs exhibited excellent viability, indicating non-cytotoxicity. The untreated control group exhibited 100% viability, while treated cells showed 98.75 ± 1.17% viability, confirming that the nanocomposites did not significantly impact normal cell survival, even at higher concentrations. These findings indicate that CS-ZnONCs are highly biocompatible and well tolerated by mammalian cells [97].

Phase-contrast microscopy further supported these results. As shown in Figure 11B, untreated Vero cells exhibited a typical spindle-shaped morphology and a uniform distribution. Cells treated with CS-ZnONCs at 12.5–200 µg/mL (Figure 11b–f) also maintained normal morphology, cell density, and adherence. No morphological abnormalities, including cell shrinkage, membrane disruption, or detachment, were observed at any concentration. The preservation of normal cellular architecture indicates that the nanocomposites did not cause structural damage. A recent study also demonstrated that NPs treatment did not induce noticeable morphological alterations in Vero cells, and cell viability remained high [98].

The biocompatibility of CS-ZnONCs is likely due to chitosan and plant-derived phytochemicals, which act as capping and stabilizing agents on the nanoparticle surface. Chitosan’s biodegradability, biocompatibility, and low toxicity help mitigate oxidative stress caused by ZnO nanoparticles [99]. Phytochemicals from plant-mediated synthesis further enhance stability and decrease cellular toxicity by preventing aggregation and limiting direct interaction with cell membranes. CS-ZnONCs exhibit negligible cytotoxicity and maintain Vero cell viability comparable to that of untreated controls. Their potential utility in biomedical applications, including medication administration, antibacterial therapy, and wound healing, is supported by their high cytocompatibility [100].

### 3.13. Wound Healing Activity

**Figure 12:**
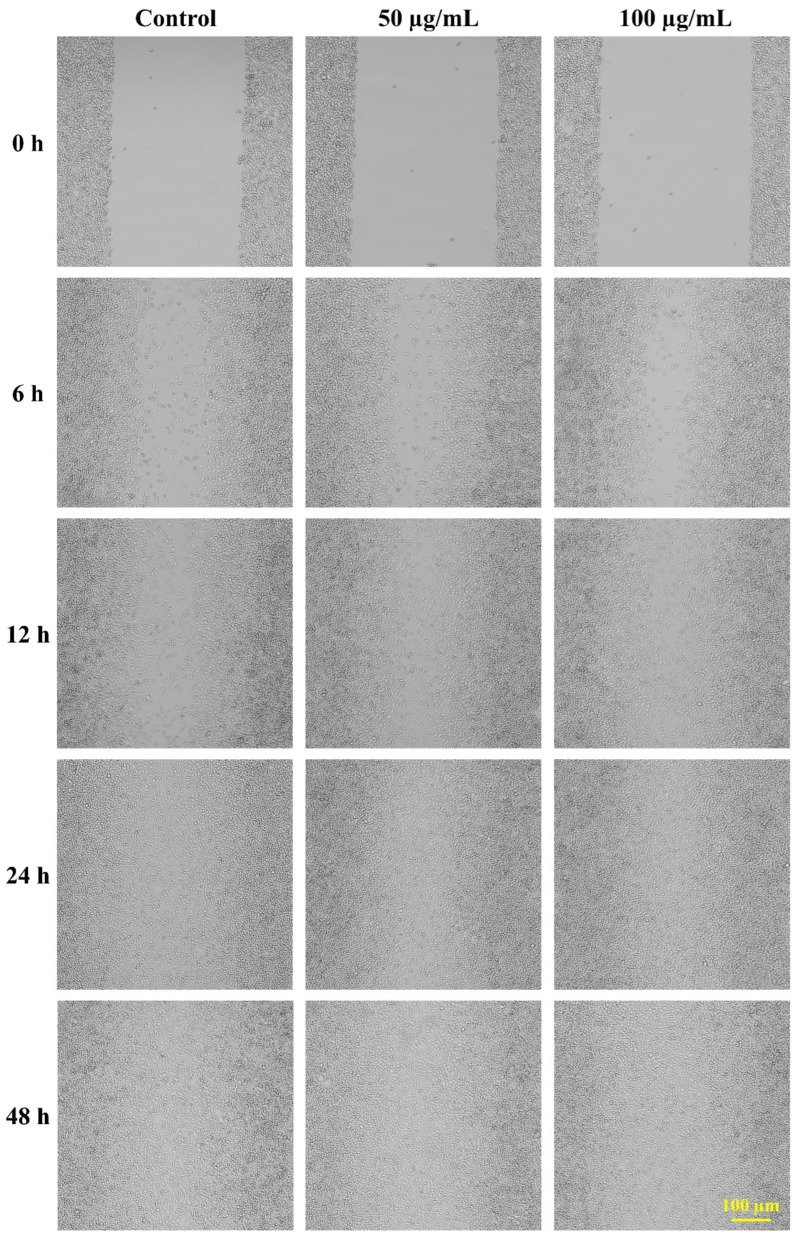
In vitro wound healing (scratch) assay showing the effect of CS-ZnONCs (50 and 100 µg/mL) on cell migration at 0, 6, 12, 24, and 48 h compared with control, demonstrating concentration- and time-dependent wound closure (scale bar: 100 µm).

Figure 12 shows the results of a scratch assay conducted on L929 mouse fibroblast cells to evaluate the in vitro wound healing ability of CS-ZnONCs. At 0 h, microscopic examination confirmed a uniform scratch across all experimental groups, ensuring consistent wound creation. In the control group, cell migration into the scratched region occurred gradually, yet wound closure remained incomplete after 48 h of incubation. On the other hand, CS-ZnONCs therapy at 50 and 100 µg/mL markedly improved wound closure, with the effect dependent on both time and concentration. At 6 and 12 h, treated cells exhibited accelerated migration toward the wound area compared to controls. By 24 h, the scratch region in CS-ZnONCs-treated groups was substantially reduced, and the 100 µg/mL treatment achieved near-complete wound closure by 48 h, while the control group retained a visible gap. Quantitative analysis of the accompanying table further demonstrates a significantly higher percentage of wound closure in CS-ZnONCs-treated cells compared to untreated controls [101].

**Figure 13:**
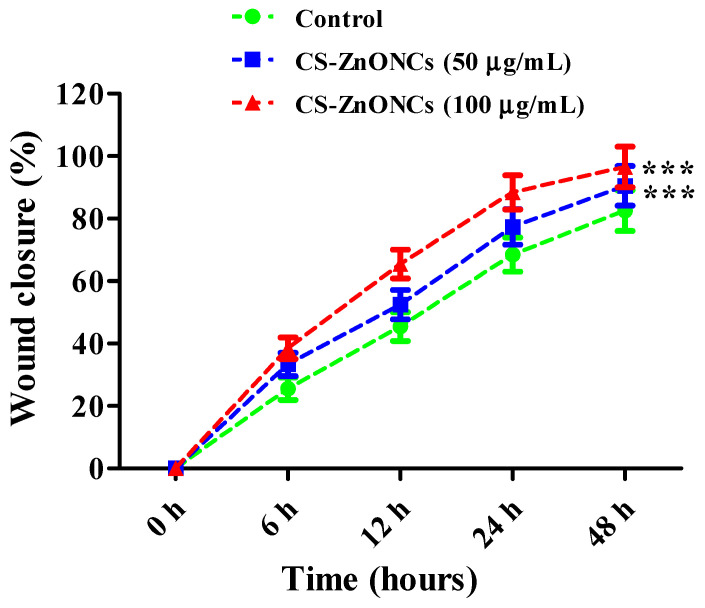
Quantitative analysis of wound closure (%) in the scratch assay showing enhanced cell migration in CS-ZnONCs–treated groups (50 and 100 µg/mL) compared with control over 0–48 h. The data are expressed as mean ± SD (n = 3); statistical significance was established at *** *p* < 0.001 when compared to the control.

CS-ZnONCs treatment resulted in significantly enhanced wound closure, with a clear time- and concentration-dependent effect. Quantitative analysis showed that the untreated control group achieved 25.56 ± 3.57% wound closure at 6 h, increasing to 45.46 ± 4.57%, 68.46 ± 5.46%, and 87.54 ± 6.46% at 12, 24, and 48 h, respectively. Treatment with 50 µg/mL CS-ZnONCs improved migration, with wound-closure values of 33.34 ± 3.75%, 52.46 ± 4.68%, 77.35 ± 5.68%, and 90.46 ± 6.35% at the corresponding time points. The 100 µg/mL CS-ZnONCs group demonstrated the most pronounced wound healing response, achieving 38.46 ± 3.46% closure at 6 h, 65.46 ± 4.57% at 12 h, 88.44 ± 5.46% at 24 h, and near-complete wound closure of 96.55 ± 6.46% at 48 h (Figure 13) [102].

The superior wound healing activity observed at 100 µg/mL is attributed to the synergistic effects of CS-ZnONCs. Chitosan promotes fibroblast adhesion, migration, and proliferation, while ZnO nanoparticles stimulate cellular growth, collagen synthesis, and growth factor expression [103]. Additionally, the nanocomposites’ anti-inflammatory and antioxidant properties reduce inflammation and oxidative stress at the wound site, thereby accelerating tissue regeneration [104]. A recent study also demonstrated that a zinc-chitosan hydrogel incorporating ellagic acid significantly improved wound-healing activity [105]. These findings collectively indicate that CS-ZnONCs effectively promote fibroblast migration and wound closure, highlighting their strong potential as wound-healing biomaterials.

## 4. Conclusions

In conclusion, this study shows that CS-ZnONCs were successfully synthesized in a green manner using *L. aspera* leaf extract as a reducing and stabilizing agent. Comprehensive physicochemical characterization demonstrated the formation of nanosized, crystalline ZnO uniformly incorporated into the chitosan matrix, exhibiting improved dispersion, stability, and surface functionality for potential biomedical applications. CS-ZnONCs demonstrated excellent swelling capacity and pH-responsive sustained drug release, confirming their suitability as effective controlled drug delivery. Antioxidant assays (DPPH, ABTS, and H_2_O_2_) revealed significant, concentration-dependent free radical scavenging activity of CS-ZnONCs. The nanocomposites demonstrated strong antibacterial activity against various bacteria, mediated by membrane disruption, enzyme inactivation, DNA damage, and ribosomal disassembly. The anti-inflammatory activity showed significant protection against erythrocytosis, confirming CS-ZnONCs’ ability to stabilize biological membranes and suppress inflammation-related damage. The Vero cell line showed non-toxicity and significant biocompatibility. In contrast, L929 fibroblast cells used in an in vitro scratch assay for wound healing exhibited rapid cell migration and near-complete wound closure, suggesting a strong regenerative capacity. Nevertheless, this study has some limitations, and more in vivo research is needed to verify the safety and therapeutic effectiveness of CS-ZnONCs in the pharmaceutical system. In addition, detailed molecular investigations are needed to clarify the signaling pathways and mechanisms underlying their biological activities. Overall, the findings suggest that CS-ZnONCs are a promising, multifunctional, and biocompatible nanomaterial with potential applications in biomedical and wound management therapies.

## Figures and Tables

**Table 1:** GC–MS analysis of phytochemical compounds identified in *L. aspera* leaf extract.

S. No.	Retention Time (min)	Area (%)	Compound Name	Molecular Weight (g/mol)
1	3.646	9.89	1,1-Diethoxyethane	90.12
2	4.107	8.67	3-Methyl-1-butanol	88.15
3	4.152	2.04	3-Methyl-1-butanol	88.15
4	4.530	1.43	2(3H)-Furanone, dihydro-3-methylene-	112.13
5	4.595	3.73	(S)-(+)-2-Pyrrolidinemethanol	101.15
6	4.702	2.33	Diethyl carbonate	118.13
7	7.888	49.87	Cyclopentane	70.13
8	11.508	0.53	Dodecane	170.34
9	14.345	1.35	Tetradecane	198.39
10	15.584	1.70	5-(Morpholino)pent-2-en-4-ynal	169.18
11	16.777	0.44	1,2-Benzenedicarboxylic acid, diethyl ester	222.24
12	16.858	1.68	Hexadecane	226.44
13	19.112	0.94	Octadecane	254.50
14	19.794	0.67	1,2-Benzenedicarboxylic acid, bis(2-methylpropyl) ester	278.34
15	20.413	0.71	Hexadecanoic acid, methyl ester	270.45
16	20.767	5.40	1,2-Benzenedicarboxylic acid, butyl ester	250.29
17	21.086	6.15	Hexadecanoic acid, ethyl ester	284.48
18	21.154	0.51	Eicosane	282.55
19	22.680	0.35	Octadecanoic acid	284.48
20	22.957	1.60	Octadecanoic acid, ethyl ester	312.53

**Table 2:** Antibacterial activity of synthesized CS-ZnONCs at different concentrations (10–100 µg/mL) against various pathogenic bacteria. Data are presented as mean ± SD. Statistical significance relative to the positive control is indicated as: * *p* < 0.05, ** *p* < 0.01, and *** *p* < 0.001.

Name of the Pathogens	Zone of Inhibition (mm) Concentration (µg/mL)				
	PC	10	25	50	100
*S. aureus*	22.67 ± 1.24	9.92 ± 0.54 **	12.58 ± 0.62 ***	15.49 ± 0.73 **	18.78 ± 0.98 ***
*B. cereus*	20.41 ± 1.19	8.94 ± 0.48 ***	10.12 ± 0.59 **	13.18 ± 0.68 ***	16.91 ± 0.84 **
*S. oralis*	20.15 ± 1.12	8.12 ± 0.42 **	9.83 ± 0.55 *	12.79 ± 0.61 **	15.42 ± 0.79 ***
*E. coli*	21.19 ± 1.46	7.15 ± 0.53 ***	10.95 ± 0.60 **	14.83 ± 0.84 ***	17.14 ± 0.96 ***
*S. enterica*	21.89 ± 1.10	8.63 ± 0.59 ***	10.64 ± 0.68 **	13.97 ± 0.75 ***	15.78 ± 0.91 ***
*K. pneumoniae*	20.52 ± 1.16	8.17 ± 0.60 **	9.19 ± 0.57 ***	12.28 ± 0.70 **	15.83 ± 0.89 **

**Table 3:** The MIC and MBC activity of CS-ZnONCs against selected pathogenic bacteria.

Pathogen	MIC (µg/mL)	MBC (µg/mL)
*S. aureus*	10	25
*B. cereus*	25	50
*S. oralis*	50	100
*E. coli*	25	50
*S. enterica*	50	100
*K. pneumoniae*	50	100

## Data Availability

Data are available from the authors upon request.
